# Human Infection with *Rickettsia felis,* Kenya

**DOI:** 10.3201/eid1607.091885

**Published:** 2010-07

**Authors:** Allen L. Richards, Ju Jiang, Sylvia Omulo, Ryan Dare, Khalif Abdirahman, Abdile Ali, Shanaaz K. Sharif, Daniel R. Feikin, Robert F. Breiman, M. Kariuki Njenga

**Affiliations:** Author affiliations: Naval Medical Research Center, Silver Spring, Maryland, USA (A.L. Richards, J. Jiang);; Uniformed Services University of the Health Sciences, Bethesda, Maryland, USA (A.L. Richards);; US Centers for Disease Control and Prevention, Nairobi, Kenya (S. Omulo, R. Dare, D.R. Feikin, R.F. Breiman, M.K. Njenga);; Kenya Ministry of Health, Nairobi (K.A. Abdirahman, A. Ali, S.K. Sharif)

**Keywords:** bacteria, Rickettsia felis, vector-borne infections, Rickettsia, Kenya, spotted fever group, rickettsiosis, flea-borne infections, quantitative PCR, research

## Abstract

To determine the cause of acute febrile illnesses other than malaria in the North Eastern Province, Kenya, we investigated rickettsial infection among patients from Garissa Provincial Hospital for 23 months during 2006–2008. Nucleic acid preparations of serum from 6 (3.7%) of 163 patients were positive for rickettsial DNA as determined by a genus-specific quantitative real-time PCR and were subsequently confirmed by molecular sequencing to be positive for *Rickettsia felis*. The 6 febrile patients’ symptoms included headache; nausea; and muscle, back, and joint pain. None of the patients had a skin rash.

## Abstract 2

This flea-borne pathogen was detected in febrile patients in North Eastern Province, Kenya.

Rickettsial diseases are found worldwide and are caused by infection with obligate intracellular rickettsiae, which are transmitted to humans by arthropod vectors (e.g., lice, fleas, ticks, and mites). Rickettsiae are associated with arthropods for a least a part of their life cycle and are passed to other arthropods by transovarial transmission or horizontal transmission involving vertebrate hosts (*1*). Rickettsiae are small, gram-negative, fastidious bacteria of the α subdivision of Proteobacteria, which are frequently divided into 2 groups based on antigenicity, G+C content, culture conditions, and actin polymerization: 1) the typhus group including *Rickettsia prowazekii* and *R. typhi,* the causative agents of louse-borne epidemic and flea-borne murine typhus, and 2) the spotted fever group including >20 species that may cause tick-, flea-, and mite-borne rickettsioses (*2*). Infection of humans by rickettsiae often begins at the site of the bite of the arthropod vector and subsequently spreads through the draining lymph nodes throughout the body, resulting in infection of endothelial cells, which can lead to multiorgan pathologic changes associated with potentially life-threatening diseases (*1*).

Little is known about the full spectrum of rickettsial diseases that occur in Kenya. One frequently reported rickettsial disease has been described in many parts of Kenya as Kenya fever, Kenya typhus, Kenya tick fever, or Kenyan tick typhus (*3**,**4*). This disease was initially thought to be caused by a single pathogen, *R. conorii*, the causative agent of Mediterranean spotted fever (*5*). However, recently, a second agent has been suggested as a cause of at least a portion of these rickettsioses (*6**,**7*), *R. africae*, the causative agent of African tick-bite fever. In addition to the tick-borne spotted fever disease, flea-borne murine typhus is known to occur in Kenya, especially in urban areas where the house rat (*Rattus rattus*) is prevalent (*4**,**8*). However, louse-borne epidemic typhus does not appear to be endemic to Kenya (*4*).

The objective of the hospital-based surveillance we report here was to determine the etiologic agents of nonmalaria acute febrile illnesses in the North Eastern Province, Kenya, during a period when there is no Rift Valley fever (RVF) epidemic in the country. The North Eastern Province was severely affected by the 2 most recent RVF epidemics in the Eastern Africa region; >89,000 human RVF infections occurred during the 1997–98 epidemic and >300 acute cases occurred during the 2006–2007 epidemic (*9**,**10*). Therefore, the surveillance was established to investigate whether acute RVF cases occurred during interepidemic periods. As part of this investigation, we also investigated the occurrence of other causes of fever that are not routinely tested for at the local hospitals, including rickettsiosis, brucellosis, leptospirosis, and other viral infections. The study protocol was approved by the institutional review boards of the US Centers for Disease Control and Prevention, US Naval Medical Research Center, and Kenya Medical Research Institute in compliance with all applicable regulations governing the protection of human subjects.

## Materials and Methods

### Study Site

This hospital-based study was conducted at the Garissa Provincial Hospital in Garissa town, located in North Eastern Province, Kenya (Figure). The semiarid province has an average annual rainfall <40 mm; is inhabited by a predominantly seminomadic pastoralist population of Somali origin who keep cattle, sheep, goats, and camels; and carries a wide range of wildlife, including zebras, antelopes, waterbucks, giraffes, warthogs, monkeys, gerenuks, dik diks, lions, hyenas, and cheetahs. Livestock interacts freely with wildlife, particularly with the large population of zebras, waterbucks, and warthogs with which they share pastures and watering holes. Local residents keep dogs primarily for security and for herding livestock.

**Figure:**
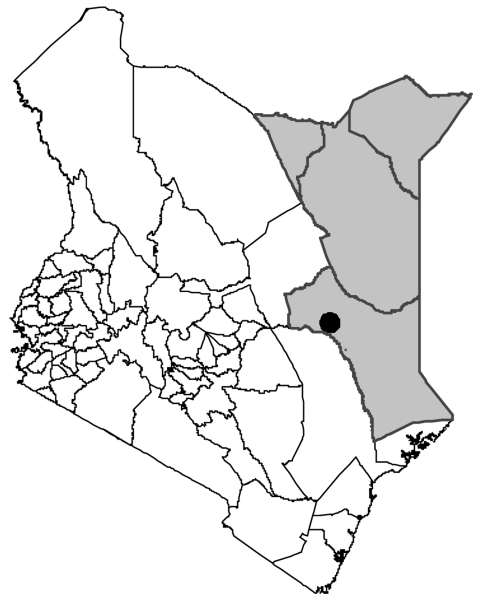
North Eastern Province (shaded area), Kenya. The city of Garissa is marked with a black circle.

### Study Participants

Rickettsial infections were evaluated as potential causes of non-malaria fever among 163 patients with fever, muscle pain, back pain, headache, and joint pain treated at the Garissa Provincial Hospital. A consenting patient was enrolled in the study if he or she was >5 years of age (inpatients or outpatients), had an axillary temperature >38°C, had a negative test for malaria by blood smear, and had a history of animal contact. Animal contact was described as herding, milking, or slaughtering. Blood samples were obtained from all consenting patients who met the criteria listed. A standardized questionnaire was administered by a clinical officer to record the patient’s clinical signs, symptoms, and potential risk factors, such as contact with animals, consumption of unpasteurized dairy products, and unsanitary sources of drinking water.

### Specimen Collection and Transportation

Approximately 3 mL of blood was collected from each patient by venipuncture using sterile technique. Serum samples were separated and stored at –20°C before being transported to the Kenya Medical Research Institute/US Centers for Disease Control and Prevention laboratory in Nairobi for acute-phase PCR and serologic testing.

### DNA Extraction

DNA was extracted from 300 µL of serum by using the QiaAmp DNA Blood Mini Kit (QIAGEN, Valencia, CA, USA). The sample DNA was first treated with 30 µL of proteinase K and then lysed with 300 µL of the kit buffer. An equal volume of absolute ethanol was added to the lysed sample and the mixture added to the DNA trapping columns provided. The columns were then freed of cell debris and other contaminants by a series of washes before releasing the matrix-trapped DNA in 50 µL of the kit elution buffer.

### Quantitative Real-time PCRs

Two quantitative real-time PCR (qPCR) assays were used to screen for rickettisal nucleic acid in the patients’ serum samples: 1) the *Rickettsia* genus–specific Rick17 qPCR that amplifies and detects a 115-bp segment of the 17-kDa antigen gene (*11*) and 2) the tick-borne rickettsiae-specific qPCR that amplifies and detects a 128-bp segment of the outer membrane protein (*ompB*) gene (*12*). The *R. felis*–specific qPCR that amplifies and detects a 129 bp segment of the *ompB* gene was also used, as previously described (*13**,**14*). The primer and probe oligonucleotides were synthesized by Sigma Genosys (The Woodlands, TX, USA). Fluorescence was monitored each cycle during the annealing step, and data were analyzed with SmartCycler software version 2.0d (Cepheid, Sunnyvale, CA, USA).

A plasmid DNA constructed with a PCR product amplified from the *R. rickettsii* 17-kDa antigen gene was used as the positive control at 10^3^ copies/reaction for the Rick17 qPCR . Plasmid DNAs constructed with the target sequences amplified from *R. rickettsii* and *R. felis ompB* were used as positive controls at 10^3^ copies/reaction for tick-borne rickettsiae-specific and *R. felis* qPCRs, respectively. Three negative controls included in each run for all qPCRs were consistently negative.

### PCR, Nested PCR, and Sequencing

Oligonucleotide primers and procedures used for PCR and nested PCR amplification of 17-kDa antigen gene, *ompB*, and *R. felis* plasmids have been described elsewhere (*15**,**16*). Briefly, the PCR products from the 17-kDa gene, *ompB*, and pRF plasmid were purified by using a QIAquick PCR purification kit (QIAGEN). No positive controls were used in these PCR and nested PCR procedures to decrease the chance of contamination; however, a single negative control was run with the samples and did not produce any products. The BigDye Terminator v 3.0 Ready Reaction Cycle Sequencing Kit (Applied Biosystems, Foster City, CA, USA) was used in the subsequent sequencing reactions according to manufacturer’s instructions. Sequencing products were purified by using Performa Gel Filtration Cartridges (EdgeBioSystems, Gaithersburg, MD, USA), and sequencing was performed on an ABI Prism 3100 Genetic Analyzer (Applied Biosystems). The primers used for PCR amplification were the same primers as those used for the sequencing reactions. At least 2 sequencing reactions were performed for each strand of DNA. Sequences were assembled by using Sequencher 4.0 (Gene Codes Corporation Inc., Ann Arbor, MI, USA), and BLAST searches were managed on the National Center for Biotechnology Information website (http://blast.ncbi.nlm.nih.gov).

## Results

A total of 163 patients whose illnesses met the case definition (fever, but not malaria) were seen at Garissa Provincial Hospital from August 1, 2006, through June 30, 2008. No patients had evidence of acute RVF disease either by PCR or immunoglobulin (Ig) M testing, but samples obtained from 35 (21.5%) patients were positive for RVF-specific IgG, indicating previous infection with the RVF virus.

To ascertain whether the patients’ serum samples contained molecular evidence of rickettsiae, DNA was extracted and the preparations were assessed by qPCRfor genus-specific 17-kDa gene (Rick17 assay) and a segment of the *ompB*, which is specific for tick-borne rickettsiae. Six samples of 163 (3.7%) tested were positive by Rick17, but none were positive by tick-borne rickettsiae–specific assay, suggesting nontick-associated rickettsiae were responsible for the patients’ fevers.

To identify which rickettsiae were responsible for the patients’ illnesses, a 434-bp fragment from the 17-kDa antigen gene was amplified from 3 of 6 Rick17-positive samples by nested PCR. Amplicons (434 bp) were produced and represented most of the open reading frame (ORF 480 bp). They were subsequently sequenced, and a BLAST search was conducted. For 3 of 6 Rick17-positive samples, amplicons were produced and assessed, and sequences of 389, 412, and 412 bp were obtained and determined to be 100% identical to that provided in GenBank for *R. felis* Cal2 (GenBank accession no. CP000054). To confirm the identity of the rickettsial agents, the 6 DNA sample preparations were assessed by an *R. felis* qPCR selective for a unique portion of *ompB*. All 6 samples were positive (Table 1). In addition, after nested PCR for a fragment of *ompB*, two 599-bp amplicons were successfully produced; the sequences of these products were 100% identical with that of *R. felis* Cal2 (GenBank accession no. CP000054). Of the 6 samples tested, the *R. felis* plasmid RF was detected only in sample 1 (GenBank accession no. CP000054), and none of the 6 samples produced an RFδ product (Table 1). Collectively, these results show that *R. felis* DNA was detected in samples from 6 patients even though the rickettsial nucleic acid concentrations were low in the serum samples assessed (cycle threshold values 36.4–44.7, data not shown).

**Table 1:** Molecular detection results and characteristics of rickettsial DNA preparations in acute-phase serum samples from 6 patients who had fever, Garissa Provincial Hospital, North Eastern Province, Kenya, 2006–2008*

Patient no.	17-kDa gene sequence	*ompB* sequence	RF plasmid
1	100% identification with *Rickettsia felis*	100% identification with *R. felis*	100% identification with *R. felis*
2	100% identification with *R. felis*	–	–
3	–	–	–
4	–	100% identification with *R. felis*	–
5	–	–	–
6	100% identification with *R. felis*	–	–

*ompB, outer membrane protein B gene. All samples were positive by Rick17 PCR and Rfelis qPCR and negative by Trick quantitative PCR (qPCR), RF plasmid, and RFδ plasmid. Rick17 qPCR is specific for the 17-kDa antigen gene of *Rickettsia* species; Trick qPCR is specific for a portion of *ompB* that is common to tick-borne rickettsiae; Rfelis qPCR is specific for a portion of *ompB* that is common to *R. felis*.

The 6 patients with positive test results for *R. felis* were 7–47 years of age. Three patients were men, 2 herdsmen and 1 farmer; 2 of the 3 female patients were housewives and 1 was a student 7 years of age. All patients reported contact with livestock animals such as cattle, sheep, goats, or camels. Although this was not captured in the questionnaire, almost all livestock owners in the region had dogs that assisted with livestock herding and security. All of the *Rickettsia*-positive patients reported having had fever for 3–8 days, as well as nausea, muscle ache, back pain, headache, and joint pain (Table 2). No patients reported skin rash, and no information about possible eschars was collected. Of the 6 patients, 5 sought care from September through February, which is the rainy season, and the other was seen in May. None of the *Rickettsia*-positive patients had evidence of jaundice, neurologic signs, bleeding, or vision impairment. Of 6 *R. felis* positive patients, 1 (16%) had IgG to RVF virus—a prevalence similar to that seen in the 163 cases (21.5%). Moreover, all 6 *R. felis* patients had negative tests for leptospirosis and brucellosis (data not shown). Though no cases of leptospirosis were identified among the patients, 14.5% of the fever patients had positive tests for brucellosis (data not shown).

**Table 2:** Clinical and demographic information of patients with flea-borne spotted fever who were treated at Garissa Provincial Hospital, North Eastern Province, Kenya, 2006–2008

Patient no.	Date	Age, y/sex	Occupation	Clinical signs and symptoms	Fever duration, d	Animals with which patient had contact
1	2006 Sep 6	47/M	Herdsman	Back pain, headache, joint pain, appetite loss, nausea	8	Cattle, sheep, goats, dogs
2	2007 Oct 19	7/F	Student	Muscle aches, back pain, headache, joint pain	7	Unknown*
3	2007 Oct 30	38/F	Housewife	Muscle aches, back pain, headache, joint pain, chills, malaise, nausea	3	Cattle, sheep, goats, dogs
4	2007 Dec 10	20/M	Herdsman	Cough, back pain, headache, joint pain	3	Cattle, sheep, goats, dogs
5	2008 Feb 28	35/M	Farmer	Cough, muscle aches, back pain, headache, joint pain, malaise, fatigue, appetite loss, dizziness	7	Unknown*
6	2008 May 2	25/F	Housewife	Muscle aches, back pain, headache, joint pain, chills, malaise, fatigue, appetite loss, vomiting, nausea	3	Cattle, sheep, goats, dogs

*Responses to the questionnaires indicated these patients came in contact with livestock; however, the responses did not list specific animals.

## Discussion

*R. felis* is the causative agent of flea-borne spotted fever, an emerging zoonotic disease with wide cosmopolitan distribution in >20 countries and 5 continents (*17*). The large dissemination of *R. felis* and thus the risk for flea-borne spotted fever can be attributed to the pervasiveness of infected arthropods worldwide, including 10 flea species (*Ctenocephalides felis, C. canis, Xenopsylla cheopis, X. brasilliensis, Pulex irritans, Archeopsylla erinacei, Tunga penetrans, Ceratophyllus gallinae, Spilospsyllus cuniculi,* and *Echidnophaga gallinacean*) (*13**,**18**–**21*), as well as mites and ticks (*22**,**23*; J. Jiang and A. Richards, unpub. data). The cat flea, *C. felis,* is considered the primary vector for *R. felis* infections because this arthropod can maintain stable infected progeny through transovarial and transtadial transmission (*24**–**26*), and antibodies against *R. felis* have been detected in animals after they have been exposed to infected cat fleas (*27**,**28*). Hosts for the infected arthropod vectors include cats, dogs, opossums, and rodents; however, no viable *R. felis* has been isolated from a vertebrate host (*17*), although *R. felis* DNA has been detected in the blood of cats (*28*), opossums (*29*), dog (*30*), rodents (*21*), and humans (*14**,**30**,**31*). However, Hawley et al. (*32*) did not detect *R. felis* DNA in cats naturally infested with *R. felis*–infected cat fleas, nor did the authors of a recent study find an association between rickettsial DNA or antibodies and the presence of fever in clinically ill cats (*33*).

The clinical signs and symptoms of flea-borne spotted fever in humans are similar to those of flea-borne murine typhus and other rickettsioses and include high fever, headache, myalgia, and rash; other manifestations occur, such as abdominal pain, nausea, vomiting, cough, eschar, photophobia, and hearing loss, occur less frequently (*17**,**18**,**31**,**34**,**35*). All of the 6 patients we described with flea-borne spotted fever reported fever, headache, and back and joint pain; 4 reported muscle aches; and 3 reported nausea and appetite loss. However, none reported rash.

Because of the nonspecific signs and symptoms of flea-borne spotted fever, the disease is difficult to diagnose clinically. Molecular diagnostics, such as qPCRs, are the methods of choice for detecting *R. felis* and diagnosing flea-borne spotted fever because *R. felis* has yet to be cultured from clinical samples and because serologic assays to detect rickettsia-specific antibodies take 1–2 weeks after onset of disease to reach detectable levels (*17**,**26**,**35*). qPCRs have detected rickettsial nucleic acid in various clinical specimens (*14**,**15**,**31*). Because of the obligate intracellular nature of rickettsiae, clinical samples that include more infected cells, such as tissue biopsy samples, peripheral blood mononuclear cells, and buffy coats, will increase the likelihood of obtaining a positive result even after patients have received proper treatment with antimicrobial drugs (*15**,**36*). For this investigation, we used qPCR screening assays, PCR and multilocus sequence typing, and a species-specific qPCR algorithm to assay acute-phase serum samples to determine the rickettsial agent causing fever if 6 of 163 patients as *R. felis*. This algorithm, derived initially from the methods of Fournier et al. (*37*), has been used successfully to identify the rickettsial agents in human clinical samples (*14**,**15*).

We report the detection and clinical signs and symptoms of *R. felis* infection of humans in Kenya. Notably, the agents for the rickettsiosis were not *R. conorii, R. africae*, or *R. typhi*, agents previously known to cause rickettsial disease in Kenya. Identification of flea-borne spotted fever and its agent *R. felis* has been reported sporadically in other locations in Africa, e.g., Democratic Republic of Congo (*20*), Tunisia (*38*), Algeria (*39*), Gabon (*40*), and Egypt (*14*). Therefore, flea-borne spotted fever needs to be included in the differential diagnosis of febrile diseases in Kenya.

We identified illness caused by *R. felis* in 6 (3.7%) of 163 fever patients in a 23-month period. This number of patients in 1 portion of the country during this short period contrasts with a total of only 68 patients known worldwide (*17*). This finding suggests that risk factors associated with flea-borne spotted fever in North Eastern Province, Kenya, exist and that civilians, military personnel, displaced populations, and foreign travelers are threatened. Therefore, healthcare providers in Kenya and in countries in which travelers return from Kenya should be aware of the possibility that febrile patients might be infected with *R. felis*.

It is also of interest that 1 of 93 patients was found in the first year of the study (August 2006–July 2007), whereas 5 of 70 patients were found in the subsequent 11 months (August 2007–June 2008). Why the number of patients increased in the two ≈1-year periods is unknown. Ongoing surveillance should determine whether this increase suggests *R. felis* infection is an emerging disease or is an endemic disease just now being recognized. In addition, future studies of rickettsioses in Kenya should include an in-depth assessment of the pathogenesis, prevalence, incidence, and risks associated with flea-borne spotted fever.
